# Differential ABA sensitivity of superior and inferior rice grains is linked to cell cycle entry into endoreduplication

**DOI:** 10.3389/fpls.2025.1585022

**Published:** 2025-05-20

**Authors:** Anil Kumar Nalini Chandran, Larissa Irvin, Balpreet K. Dhatt, Yuvraj Chopra, Steven McArtney, Marci A. Surpin, Sriram K. Reddy, Harkamal Walia

**Affiliations:** ^1^ Department of Agronomy and Horticulture, University of Nebraska-Lincoln, Lincoln, NE, United States; ^2^ Valent BioSciences LLC, Libertyville, IL, United States

**Keywords:** rice, ABA, BA, grain filling, cell cycle, spikelet, yield, transcriptome

## Abstract

Suboptimal grain filling in rice (*Oryza sativa*) inferior spikelets poses a constraint to maximizing the yield potential. The differential grain filling between superior and inferior spikelets has been primarily attributed to differences in endogenous phytohormone levels that determine grain sink capacity. In this study, we aimed to gain molecular insights into the role of two phytohormones, abscisic acid (S-ABA or ABA) and cytokinin (6-benzyladenine or BA) through exogenous applications on superior and inferior grains in rice. We found that ABA and a combination of ABA and BA (ABA+BA) applications increased the grain yield in field studies, primarily by improving the grain weight of both superior and inferior grains. Transcriptomic analysis of developing grains shows differences in the expression of core cell cycle genes between the superior and inferior grains at four days after fertilization between the control and phytohormone applications. ABA and ABA+BA applications induce DNA replication genes and cell cycle inhibitory genes in superior grains only, likely promoting endoreduplication for increased cell storage capacity. ABA and BA applications suppressed the expression of cytokinin signaling genes in superior grains but induced them in inferior grains emphasizing the key roles for cytokinins and ABA in superior and inferior grains, respectively. An early induction of several grain storage-related genes in inferior grains is associated with accelerated entry into the grain storage stage, thus limiting sink capacity and poor grain fill. Our results indicate that ABA alone promotes photosynthate remobilization into both superior and inferior grains while ABA + BA regulates grain filling via cell cycle-related transcriptomic changes. Overall, our study reveals an intrinsic difference in ABA+BA sensitivity between inferior and superior grains that is linked to regulation of cell cycle checkpoints and entry into endoreduplication in the endosperm.

## Introduction

An increase in global demand for food crops necessitates a substantial increase in rice (*Oryza sativa*) production, as it is a major caloric source for humans. However, similar to other major food crops such as wheat and maize, rice yields have plateaued in recent years, even in major rice-producing regions such as South Asian countries (Bin Rahman and Zhang, 2023). This problem is further compounded by diminishing arable land and unpredictable weather patterns (Zhang and Cai, 2011; Rezvi et al., 2023). Genetic studies revealed several novel loci for improving sink strength by augmenting the number of grains per panicle and grain weight (Mao et al., 2010; Fang et al., 2016; Lu et al., 2022; Li et al., 2023). Besides enhancement of sink strength, a better understanding of grain-filling mechanisms is vital for crop improvement initiatives, because carbon partitioning and subsequent starch and other storage accumulation directly impact the yield and quality of rice grains (Wang et al., 2015a; Ma et al., 2023).

In rice, grain filling is not uniform along the length of the panicle. Spikelets that fertilize earlier fill faster and have greater grain weights. These grains are typically located on apical primary branches and are termed superior spikelets (SS) (Sun et al., 2015; Wang et al., 2019). In contrast, the later-fertilizing inferior spikelets (IS) are generally located on proximal secondary branches and are often characterized by poor grain filling rates, lower grain weights, and diminished quality, making it a substandard grade for human consumption. The molecular basis of variation in grain filling between IS and SS is not fully understood. A better understanding of the differences between SS and IS can enable improvement in rice production and quality through advanced agronomic practices and the introduction of new traits.

Phytohormones are important regulators of the grain filling mechanism and have been examined extensively for their role in grain filling and differential outcomes for superior and inferior grains in rice (Teng et al., 2022; reviewed in Ma et al., 2023). Among these, indole-3-acetic acid (IAA), ABA and BA levels are associated with grain filling rates at different points during development (Zhang et al., 2009; Yang et al., 2016). Both ABA and cytokinin accelerate carbon remobilization to enhance grain filling by controlling senescence when plants are subjected to water stress (Yang et al., 2003). Comparative analysis of wheat cultivars showed that ABA mediates transcriptomic changes associated with superior adaptation and maintaining higher yields under water deficit conditions (Reddy et al., 2014). A QTL regulating endogenous levels of these phytohormones has been shown to have a pronounced impact on grain yield. For instance, cytokinin oxidase/dehydrogenase (*OsCKX2*) degrades cytokinins, and its suppression enhances grain yield primarily through cytokinin accumulation in the inflorescence meristem (Ashikari et al., 2005). Besides lower concentrations of these phytohormones, the suboptimal grain filling in IS has also been associated with variation in endogenous phytohormone levels, status of sucrose-to-starch metabolic enzymes, nitrogen metabolism, endosperm cell division, and weakening of photosynthesis and respiration processes (Zhu et al., 2011; Li et al., 2018; You et al., 2016). The dry weight of developing grains positively correlates with the number of endosperm cells and is higher for SS than IS (Zhang et al., 2016). The rate of cell division is regulated by cytokinin levels, which peak earlier in SS compared to IS, highlighting the role of cytokinin in determining grain capacity (Zhang et al., 2010; Yang et al., 2016). Besides the timing of peaks in phytohormone levels, the IS and SS also differ in the level to which the phytohormones peak.

An inherent higher concentration of ABA promotes endosperm cell division and accelerated grain filling, leading to heavier grains in SS. Conversely, higher ethylene levels, coupled with lower levels of ABA, drive slower endosperm cell division and lead to poor grain filling in IS (Yang et al., 2006a). An increase in grain weight in IS resulting from higher cell division, cell numbers, and grain filling upon ABA application affirms its positive role in grain development (Yang et al., 2006b). Besides the imbalance in phytohormone levels, the weaker metabolism of photoassimilates, marked by lower activities of UDP-glucose pyrophosphorylase, ADP-glucose pyrophosphorylase, starch synthase, and starch branching enzymes, also contributes to poor grain filling in IS (Dong et al., 2014; You et al., 2017; Chen et al., 2019). For instance, improved grain filling in IS under moderate soil drying during the post-anthesis stage has been shown to be linked to elevated levels of ABA and IAA, along with increased activities of starch synthesis enzymes (Teng et al., 2022). This ABA-dependent yield increase is also supported by reports from wheat where ABA application increased total starch and grain weight (Yang et al., 2014). Furthermore, the relative ratios of multiple phytohormones have also been associated with grain filling. A higher ratio of ABA:ethylene promotes grain filling in rice (Yang et al., 2006a). Ethylene regulates endosperm cell division rates and the concentration differences between SS and IS are more pronounced in large-panicle rice cultivars (Sekhar et al., 2015). Changes in the concentrations of IAA and salicylic acid also have been shown to be involved in enhancing grain weight and starch content (Zhang et al., 2016; Chen et al., 2017).

Two important factors that determine grain weight, sink capacity and endosperm cell division, are primarily attributed to the regulation of mitotic cell division (Wang et al., 2019). For instance, cytokinin acts during the G1 to S and G2 to M cell cycle transitions, where its degradation causes meristematic defects (Schaller et al., 2014). Transcriptome profiling of IS and SS capturing a dynamic change in the expression of cell cycle-related genes showed prolonged activities of cyclins in IS that peaked 16–20 days after fertilization (DAF), suggesting that IS cells continue to actively divide in later stages of grain development (Schaller et al., 2014; Sun et al., 2015). Besides the mitotic divisions, activities of specific cyclins, cyclin-dependent kinases, and MYB transcription factors at the G2 to M phase transition determine whether the cell cycle progression switches to endoreduplication (Sanchez et al., 2012; Kobayashi, 2019). Endoreduplication, an event characterized by an increase in cell ploidy level and endosperm cell size, is regulated by cytokinin levels (Kobayashi, 2019). Although the effect of exogenous application of ABA and cytokinin has been investigated previously, understanding of the combinatorial effect of these phytohormones on SS and IS grain development and the underlying regulatory genes is limited. Therefore, insight into the interplay between grain-filling genes and cell cycle regulators in the context of ABA and cytokinin levels can be useful in developing strategies to enhance grain filling in IS.

Commercial use of plant growth regulators (PGRs) in the horticultural industry to improve fruit set, size, shape, and quality, for crop load management, and as a harvest management tool is widely prevalent. For example, *S*-ABA is registered to improve red table grape coloration and 6-BA is registered for fruit thinning in apple and pears. The adoption of PGRs for field crops has been slower, but nonetheless has been gaining momentum recently. For example, 6-BA for improved branching in soybeans, is registered in Brazil, and ABA-based products are being commercialized for improving grain yield and stress tolerance in rice, wheat, barley, corn and soybean.

In this study we sought to examine the physiological and genetic changes induced by exogenous application of ABA and ABA+ BA in SS and IS during grain filling via field trials and transcriptome analyses. We show that ABA and ABA+BA application improved the total grain yield in field studies, which we attribute to an increase in the grain filling in both superior spikelets and inferior spikelets. At the molecular level, cell cycle regulation and entry of cells into endoreduplication is evident as a mechanism contributing to the increased grain filling in superior grain in response to elevated levels of ABA and cytokinin, in addition to change in sink strength. We further show that cytokinin signaling genes have contrasting responses to phytohormone treatments in superior and inferior grains.

## Materials and methods

### Plant material and growth conditions

We used the long-grain tropical japonica rice variety *Katy* for the present study. This variety was selected because of its remarkable combination of disease resistance and suitable agronomic traits. Plants were grown in pots (4-inch square) under controlled greenhouse conditions, 16h light and 8h dark at 28 ± 1 °C and 23 ± 1°C, respectively, with a relative humidity of 55-60%. The plants were tracked for precise developmental progression (Folsom et al., 2014). For a primary panicle, fertilization of florets is generally completed in 4–5 days. Florets that flowered on the first two days were considered part of the SS, while those that flowered on the last two days were labeled as IS (Yang et al., 2006b). Beginning the anthesis of the primary panicle, florets were marked on the fertilization day and 4th day was the day of last marking (DLM), when the whole panicle completed flowering. The primary panicles bearing marked SS and IS were exogenously treated with phytohormones.

### Abscisic acid and 6-benzyladenine treatments on the primary panicle

Phytohormone treatments on the panicle were conducted after the entire panicle marking was completed. For the phytohormone treatments, formulations of (S)-ABA (ProTone, Valent BioSciences LLC), and the cytokinin 6-benzyladenine (MaxCel, Valent BioSciences LLC), were applied to the primary panicles. In each experiment we applied water sprays as control treatments, or 150 mg L^-1^ solution of ABA, or a solution of 150 mg L^-1^ ABA plus 1.58 ml L^-1^ 6-BA (solution A). All three batches of plants were grown under the same conditions. All solutions contained the surfactant Latron at final concentrations of 0.025% (v/v). The same volume of deionized water containing 0.25% surfactant was applied to control plants. Each chemical treatment at a given time point had at least three replicates (three plants). Solutions were delivered to the panicle as a fine mist from a spray pump at the rate of 3–4 ml per panicle.

### Field studies

Field trials testing foliar the application of ABA and ABA+BA were conducted during the 2020 dry cropping season at Bgy Sto. Tomas, San Jose City, Nueva Ecija- Central Luzon region of the Philippines. Paddy rice cultivar NSIC Rc 218 with a maturity of 121–127 days was used in transplanted field trials in a randomized complete block design with six replications. Each plot dimension was 8 m x 10 m (80 sq. meters), and 5 m x 8 m (40 sq. meters) was harvested for yield and yield component analysis (at 14% moisture content). Foliar applications were made using a battery-powered knapsack sprayer at the early grain filling stage (watery to milk stage), corresponding to about 72 d after transplanting. ABA was applied at 6 g/ha (20% active ingredient) and BA at 316 ml/ha (1.9% active ingredient) in a spray volume of 192 liters/ha. The crop production was carried out using typical grower practices from seeding and transplanting to harvest, including standard fertilizer and crop protection applications.

### RNA extraction and transcriptome analysis

We generated libraries for RNASeq from developing grains that were treated with exogenously applied ABA, ABA+BA and a control set (three plants per replicate) to study the transcriptomic changes resulting from phytohormone-mediated grain size and weight differences. Samples were collected at 2 DLM, 7 DLM and 10 DLM, corresponding to 4 DAF, 9 DAF and 12 DAF, respectively, from superior and inferior grains. Samples were collected and frozen in liquid nitrogen. These frozen samples were homogenized by cryo-cracking in liquid nitrogen using pestle and mortar. RNA was extracted from homogenized samples using the RNAeasy extraction kit according to the manufacturer’s protocol (Qiagen, Hilden, Germany). After the cDNA synthesis, RNAseq libraries were prepared using Illumina’s TruSeq Stranded mRNAseq Sample Prep kit (Illumina). The sequencing was performed using the Illumina NovaSeq 6000 sequencing system. Fastq files were generated and demultiplexed with the bcl2fastq v2.20 Conversion Software (Illumina). The quality of the raw reads was assessed using FastQC. After removing the raw reads, filtered reads were mapped to rice genome annotation v. 7 using hisat2 aligner (Kim et al., 2019). Reads mapped to exonic regions were extracted from BAM files using the featureCounts read summarization tool (Liao et al., 2014). RNA-seq gene expression values were variance-stabilized using the vst function from the DESeq2 package. The variance-stabilized dataset was used to perform PCA using the prcomp function in R (Love et al., 2014). The PCA plot was generated using ggplot2 (Wickham, 2008). The differential expression analysis was estimated using the DESeq2 package. In DESeq2, pairwise comparisons of the samples were performed using the contrast function to specify comparisons between conditions. Differentially expressed genes (DEGs) were identified with the following cutoffs: >1 fold change and FDR < 0.05. For the downstream analyses, clustering was performed using the Heatmap function from the ComplexHeatmap package (Gu, 2022).Gene ontology (GO) terms of the clusters were identified using g:Profiler tool (Kolberg et al., 2023).

## Results

### ABA and ABA+BA applications improve grain filling

**Figure 1:**
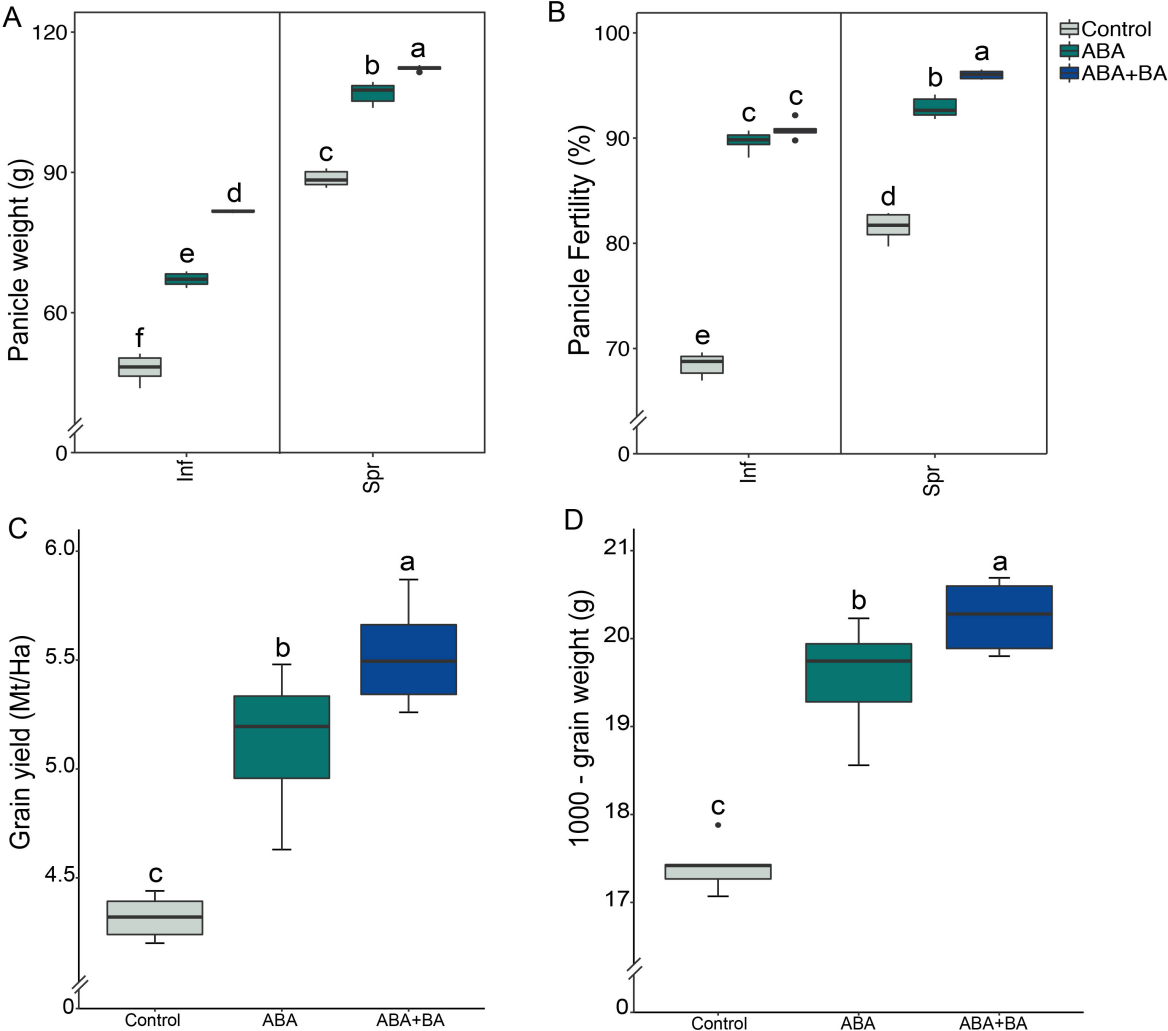
Evaluation of the impact of exogenous application of ABA and ABA+BA in superior and inferior grains in a field-based study conducted during the 2020 dry-cropping season at Bgy Sto. Tomas, San Jose City, Nueva Ecija- Central Luzon region of the Philippines. Paddy rice cultivar NSIC Rc 218 was used in transplanted field trials in a randomized complete block design with six replications. The phytohormone applications significantly improved the grain weight of **(A)** both superior and inferior panicles **(B)** Panicle fertility (%) **(C)** Total grain yield and **(D)** 1000-gram weight. Statistical significance was calculated using two-way ANOVA. Mt/Ha, Metric ton/Hectare; Inf, Inferior; Spr, Superior. Alphabets are significance leves in ANOVA test.

To evaluate the impact of the exogenous application of ABA and ABA+BA on superior and inferior grain development and yield parameters, we conducted a field study using the cultivar NSIC Rc 218. We found that ABA application significantly increased superior and inferior panicle weight compared to the controls (**Figure 1A**). Under control (non-spray) conditions, inferior and superior panicles had a mean weight of 48 and 88.6g, respectively. However, panicle weight was increased to 67.1g in inferior and 106.9g in superior panicles in response to ABA application. Notably, maximum weights for both panicle types were attained in response to ABA+BA application (mean inferior=81.6g; mean superior=112.2g) (**Figure 1A**). Application of ABA and ABA+BA also improved the spikelet fertility rate for both superior and inferior panicles, wherein fertility (%) was increased from 68.4% to 90.8% in inferior and from 81.5% to 96% in superior panicles that were sprayed with ABA+BA (**Figure 1B**). The increased panicle weight and improved spikelet fertility also resulted in increased total grain yield and 1000-grain weight (**Figures 1C, D**).

### Exogenous application of ABA and AB+BA causes extensive transcriptomic reprogramming

**Figure 2:**
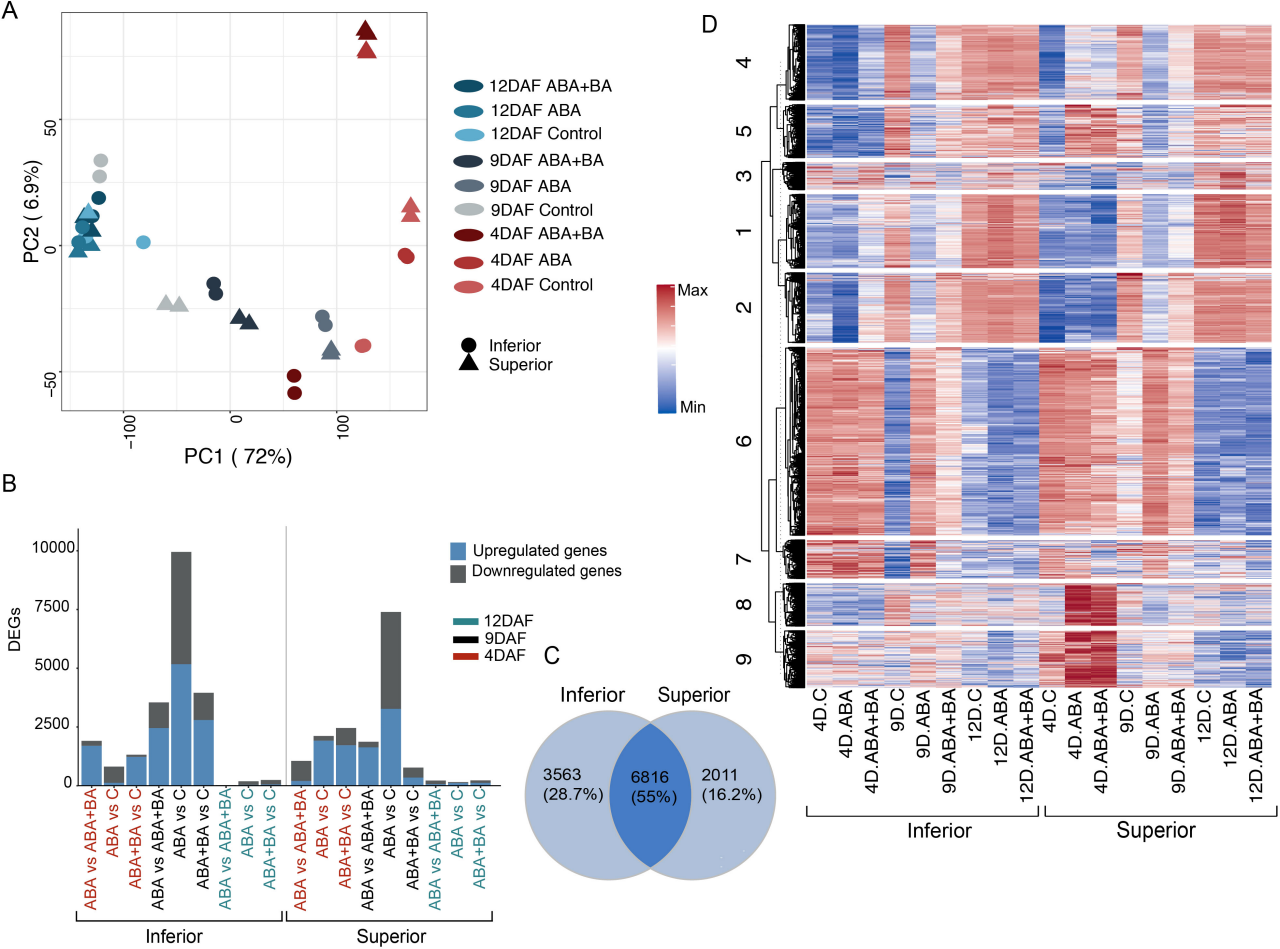
Transcriptome analysis of superior and inferior grains at 4 days after fertilization (DAF), 9DAF and 12DAF **(A)** Principal component analysis shows that samples collected 4DAF had maximum separation between superior and inferior grains, while 12DAF samples had relatively less transcriptional changes **(B)** Distribution of differentially expressed genes (DEGs) for each pairwise comparison **(C)** Venn diagram showing the overlap between superior and inferior grain DEGs **(D)** Clustering analysis of 7,362 DEGs.

Given the increase in panicle weight of both superior and inferior panicles and total grain yield, we next sought to understand the molecular basis of improved grain filling in response to ABA and ABA+BA application. We conducted a time-series transcriptomic analysis of superior and inferior grains from primary panicles grown under greenhouse conditions. To capture the transcriptome dynamics of early and mid-stages of grain development in superior and inferior grains, we applied the phytohormones (ABA and ABA+BA) and mock (control) treatments at 2, 7 and 10 DAF and collected developing grains two days later, at 4 DAF, 9 DAF and 12 DAF (**Supplementary Figure 1**). To determine the sample relationships, we performed a principal component analysis (PCA). PCA analysis separated the superior and inferior grain samples into different clusters for the control, ABA, and ABA+BA treatments (**Figure 2A**). The transcriptomes at 4 and 9 DAF exhibited greater separation in all conditions, whereas we observed a relatively smaller sample separation at 12 DAF. This analysis suggests that at the molecular level, developing superior and inferior grains differ more in the 4–9 DAF developmental window compared to the 12 DAF stage.

We next performed multiple pairwise comparisons of transcriptome samples obtained from ABA and ABA+BA applications with respect to their respective controls in superior and inferior samples at each time point. Our analysis yielded 3,353 DEGs at 4 DAF, 7,484 at 7 DAF, and 481 at 10 DAF in superior grains in at least one of the treatments (ABA or ABA+BA) (**Supplementary Table 1**, **Figure 2B**). Similarly, we identified 2,202 DEGs at 4 DAF, 10,334 at 7 DAF, and 332 at 10 DAF in inferior grains in at least one of the treatments. We found that 28.7% of genes (3,563 genes) were differentially regulated only in inferior grains, and 16.2% of DEGs (2,011 genes) were specific to superior grains (**Figure 2C**). About 55% of the genes (6,816 genes) were differentially regulated in both grain types. Collectively, a set of 7,362 genes were identified based on differential regulation in at least one comparison in response to phytohormone application or developmental stage. We estimated that the expression of 3,046 genes is regulated during developmental progression, hence classified as grain development genes. We selected the core set of 7,326 DEGs to examine treatment response patterns and development stage-based changes in transcript abundance.

We conducted a K-means clustering analysis of 7,362 genes (*K*=9) and identified clusters with featured expression patterns in response to ABA and ABA+BA application or developmental progression (**Figure 2D**). Cluster 9 consists of genes with higher expression at 4 DAF in superior grains than inferior grains, wherein their expression level further increases in response to ABA and ABA+BA application. A similar group of genes was identified in cluster 8 but had their expression uniquely induced by ABA and ABA+BA application at 4 DAF in superior grains. Induction of a set of genes in cluster 5 also revealed the effect of phytohormones application at 4 DAF transcriptome in superior grains. Expression of cluster 6 and 7 genes were repressed at 9 DAF in inferior grains and are attributed to their developmental regulation. The transcriptome response of a combination of ABA+BA applications is evident from genes in clusters 1 and 2 as the genes repressed upon ABA+BA application at 4 DAF in superior grains compared to inferior grains. These patterns indicate a diverse transcriptomic response between SS and IS upon phytohormone application.

### ABA+BA alters the cell cycle progression specifically in superior spikelets

**Figure 3:**
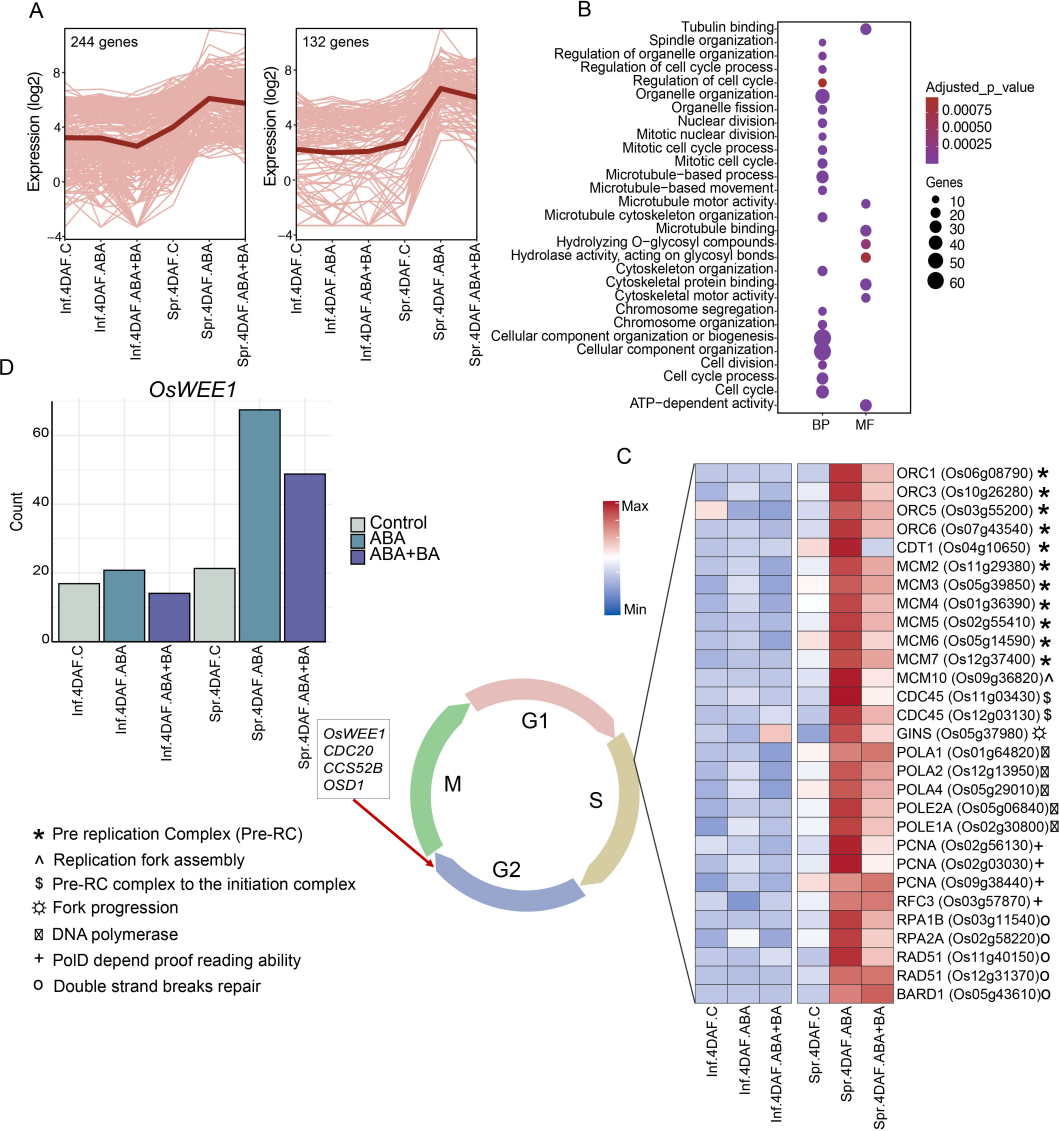
AB and ABA+BA induce changes in cell cycle regulation specifically in superior grains **(A)** Clusters of genes that show superior grain preferred expression at 4DAF under control conditions, ABA and ABA+BA applications **(B)** Gene ontology shows the enrichment of cell cycle related terms in the clusters **(C)** Activation of DNA replication machinery genes in superior grains under phytohormone applications **(D)** Induction pattern of endoreduplication regulator *OsWEE1* in superior grains under phytohormone application. BP-Biological process, MF-Molecular function, CC- Cellular component. Inf-Inferior, Spr-Superior.

We first examined the genes in clusters 5, 8 and 9 as the major difference in transcriptome profiles between superior and inferior grains was resolved from these clusters at 4 DAF (**Figure 2D**). Our analysis showed that ABA and ABA+BA application induced the expression of 132 genes in superior grains at 4 DAF, whereas these genes remained repressed in inferior grains in both controls and under phytohormone applications at the same time point (**Figure 3A**). Similarly, 244 genes, for which expression was relatively higher in superior grains in controls compared to that of inferior grains, were further induced by ABA and ABA+BA application. Our subsequent analysis showed that these two clusters prominently feature genes involved in cell cycle regulation, as several GO terms such as cellular component organization, microtubule binding, tubulin binding and cytoskeleton motor activity are overrepresented among the genes (**Figure 3B**). A differential expression of cell cycle genes indicates an inherent difference in endosperm cellularization between superior and inferior grains under control conditions, which persists under ABA and ABA+BA applications.

To get an insight into changes in cell cycle progression in the two spikelet types under ABA and ABA+BA applications, we analyzed the gene expression pertinent to distinct cell cycle phases with a focus on 4 DAF as this development stage accounts for the highest expression difference between the spikelet types. A cooperative interaction of cyclin-dependent kinases (CDKs) and cyclins (CYC), specifically the CDKA-CYCD complex, primarily drives the cell cycle progression (Gutierrez, 2009; Genschik et al., 2014). Although ABA and ABA+BA treatment alters the expression pattern of these genes, most of the CDKA-CYCD subfamily genes (except *CYCD6;1*) and their phosphorylation targets, retinoblastoma-related proteins (RBRs), did not show a spikelet-type specific difference, indicating that the G1 phase genes are not differentially regulated by ABA and ABA+BA application in the two spikelet types. However, a striking difference in the cell cycle regulation between SS and IS induced by ABA and ABA+BA was observed during the G1 to S phase transition at the onset of DNA replication (**Figure 3C**). Genes encoding major pre-replication complex (RC), such as *ORC1*, *ORC3*, *ORC5*, *ORC6*, *CDT1*, *MCM2*, *MCM3*, *MCM4*, *MCM5*, *MCM6* and *MCM7*, showed a significant induction in SS in response to ABA and ABA+BA application at 4 DAF (**Figure 3C**). Our observation is also supported by the induction of genes regulating replication fork assembly (*MCM10*; *LOC_Os09g36820*), switch from the pre-RC complex to the initiation complex (*CDC45*; *LOC_Os11g03430* and *LOC_Os12g03130*), fork progression (GINS; *LOC_Os05g37980*), DNA Polymerases complex (*POLA1*; *LOC_Os01g64820*, *POLA2*; *LOC_Os12g13950*, *POLE2A*; *LOC_Os05g06840*, *POLA4*; *LOC_Os05g29010* and *POLE1A; LOC_Os02g30800), PolD* depend proofreading ability (*PCNA*; *LOC_Os02g56130*, *LOC_Os02g03030* and *LOC_Os09g38440*), RFC3 replication factor subunit (*LOC_Os03g57870*), RPA, that is required for pre-RC activation and loading of essential initiator factors at origins (*RPA1B*; *LOC_Os03g11540*, *RPA2A*; *LOC_Os02g58220*) and DNA repair proteins (*RAD51*; *LOC_Os11g40150* and *LOC_Os12g31370*, *BARD1*; *LOC_Os05g43610*). The upregulation of S-phase genes shows that ABA and ABA+BA application induces differential regulation of DNA replication machinery in superior grains.

**Figure 4:**
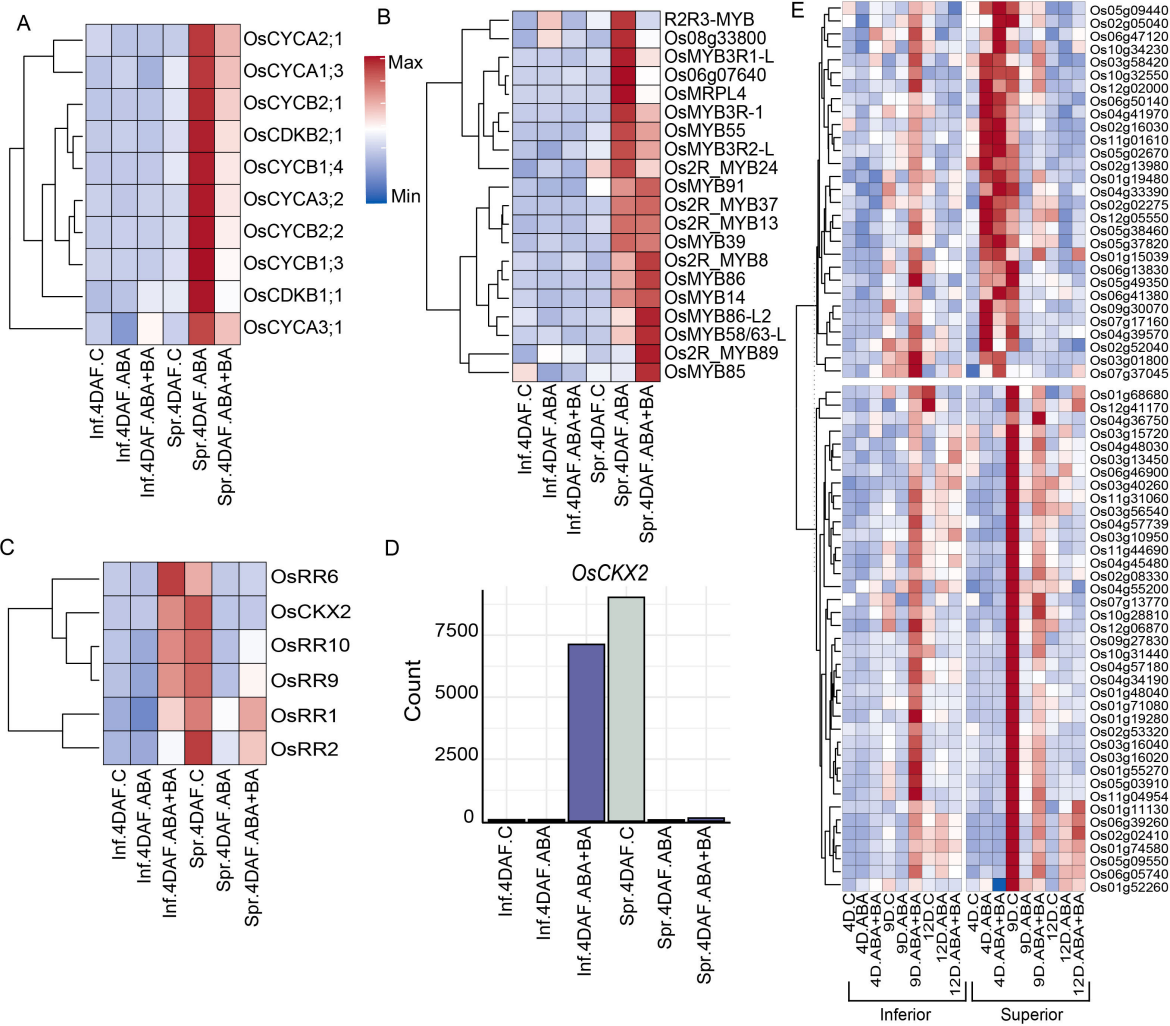
Phytohormone application showed superior grain-specific induction of a group of cyclins (CYC), MYB and cytokinin inhibitor genes **(A)** Induction of CYC, and CDKB **(B)** MYB family genes **(C)** Differential regulation of cytokinin inhibitory genes in superior and inferior grains **(D)** Induction pattern of cytokinin dehydrogenase (*OsCKX2*) in superior and inferior grains. **(E)** Clusters of genes responsive to ABA+BA treatment. Inf, Inferior; Spr, Superior.

Even though a clear difference in the expression of G1-phase genes between SS and IS was not observed, we found that ABA and ABA+BA induced expression of two putative CDKA-CYCD inhibitors (CKIs) kinase inhibitory proteins (KIPs) (KIP1; *LOC_Os03g43684* and *LOC_Os12g41200*) and SIAMESE-RELATED gene and its upstream target (*SMR4*; *LOC_Os01g62584* and *SMOS1*; *LOC_Os05g32270*, respectively). Given that the induction of these two classes of CKIs is likely to decrease CDK activity together with activation of DNA replication machinery, we speculate that ABA and ABA+BA application induces the cells to undergo endoreduplication specifically in superior grains similar to the pattern identified in Arabidopsis, where KIP and SIAMESE are required to suppress mitosis and the switch to the endoreduplication (Verkest et al., 2005; Churchman et al., 2006). To examine whether cells in developing superior grains possibly switch to an endoreduplication state, we analyzed the expression of key genes that mediate the endocycle and found a similar induction pattern for *OsWEE1* (*LOC_Os02g04240*), as its elevated expression controls the endocycle and leads to cell expansion by negatively regulating CDK activity (Gonzalez et al., 2007)(**Figure 3D**). A similar induction pattern was also found for co-activators of anaphase promoting complex/cyclosome (APC/C) (*CDC20*; *LOC_Os02g47180* and *CCS52B*; *LOC_Os01g74146*), which degrades CYCs and controls the endocycle switch. The APC/C-dependent regulator of the endocycle, *OSD1* (*LOC_Os02g37850*) also exhibits a similar expression pattern in SS. In a previous report involving Fly Base mutants, it has been shown that CYCA and CYCB proteins accumulate during endoreduplication (Sallé et al., 2012). In line with this observation, ABA and BA+BA induced four CYCAs (*CYCA3;2*; *LOC_Os03g41100*, *CYCA2;1*; *LOC_Os12g31810*, *CYCA3;1*; *LOC_Os12g39210* and *CYCA1;3*; *LOC_Os01g13260*), four CYCBs (*CYCB2;1*; *LOC_Os04g47580*, *CYCB2;2*; *LOC_Os06g51110*, *CYCB1;3*; *LOC_Os05g41390* and *CYCB1;4*, *LOC_Os01g59120*), *CDKB1;1* (*LOC_Os01g67160*) and *CDKB2−1* (*LOC_Os08g40170*) specifically in SS at 4 DAF, further supporting the hypothesis that superior grains switch to endoreduplication (**Figure 4A**).

In addition to the core cell cycle genes, we also found the induction of several transcription factors (TF) in phytohormones-treated samples in a similar pattern. Among the family of genes that regulate the G2-to-M phase transition, we found the induction of 20 MYB family genes in superior grains that were treated with ABA or ABA+BA at 4 DAF (**Figure 4B**). Differential regulation of cell cycle genes and potential genes associated with the endoreduplication switch due to phytohormones application prompted us to investigate the factors that influence changes in cell cycle progression. We therefore analyzed the DEGs between superior and inferior grains at 4 DAF under control conditions and showed that inferior grains normally have lower expression of genes that repress the cytokinin content relative to superior grains, indicating that inferior grains have higher endogenous cytokinin levels (**Figures 4C, D**). We found that ABA+BA application induced the cytokinin inhibitory genes *OsRR1*, *OsRR2*, *OsRR6*, *OsRR9* and *OsCKX2* in inferior grains, whereas these genes are suppressed by ABA and ABA+BA application in superior grains. These observations suggest that cytokinin levels may be one of the critical factors in ABA-induced cell cycle regulation in superior grains.

To determine the impact of ABA+BA treatment relative to ABA treatment, we sought to determine the genes that are largely responsive to ABA+BA. We found that ABA+BA specifically induced 39 genes in inferior spikelets at 9 DAF (**Figure 4E**). In contrast, superior spikelets had a higher basal expression for these genes and ABA+BA suppressed their expression wherein both inferior and superior had a comparable expression level. Similarly, the expression of 29 genes was induced only in response to ABA+BA in the inferior spikelets at 9 DAF. These genes were induced at an earlier time point (4 DAF) in both ABA and ABA+BA treatments in superior spikelets. These genes were suppressed at 9 DAF in response to both treatment types. Among the genes responsive to BA, *OsDMC1* (*LOC_Os11g04954*) is involved in meiotic division and plays a vital role in synapsis (Wang et al., 2016). Cytokinin (CK) dehydrogenase precursor *OsCKX3* (*LOC_Os10g34230*) maintains the CK homeostasis and regulates cell proliferation controlling 1,000-grain weight (Huang et al., 2023). These expression patterns indicate that the upregulation of genes in response to ABA+BA treatment leads to an increase in the grain weight of inferior spikelets. Moreover, BA fine-tunes the expression of several ABA induced genes in superior spikelets in order to attain maximum yield potential.

### ABA and ABA+BA applications regulate grain-filling genes

**Figure 5:**
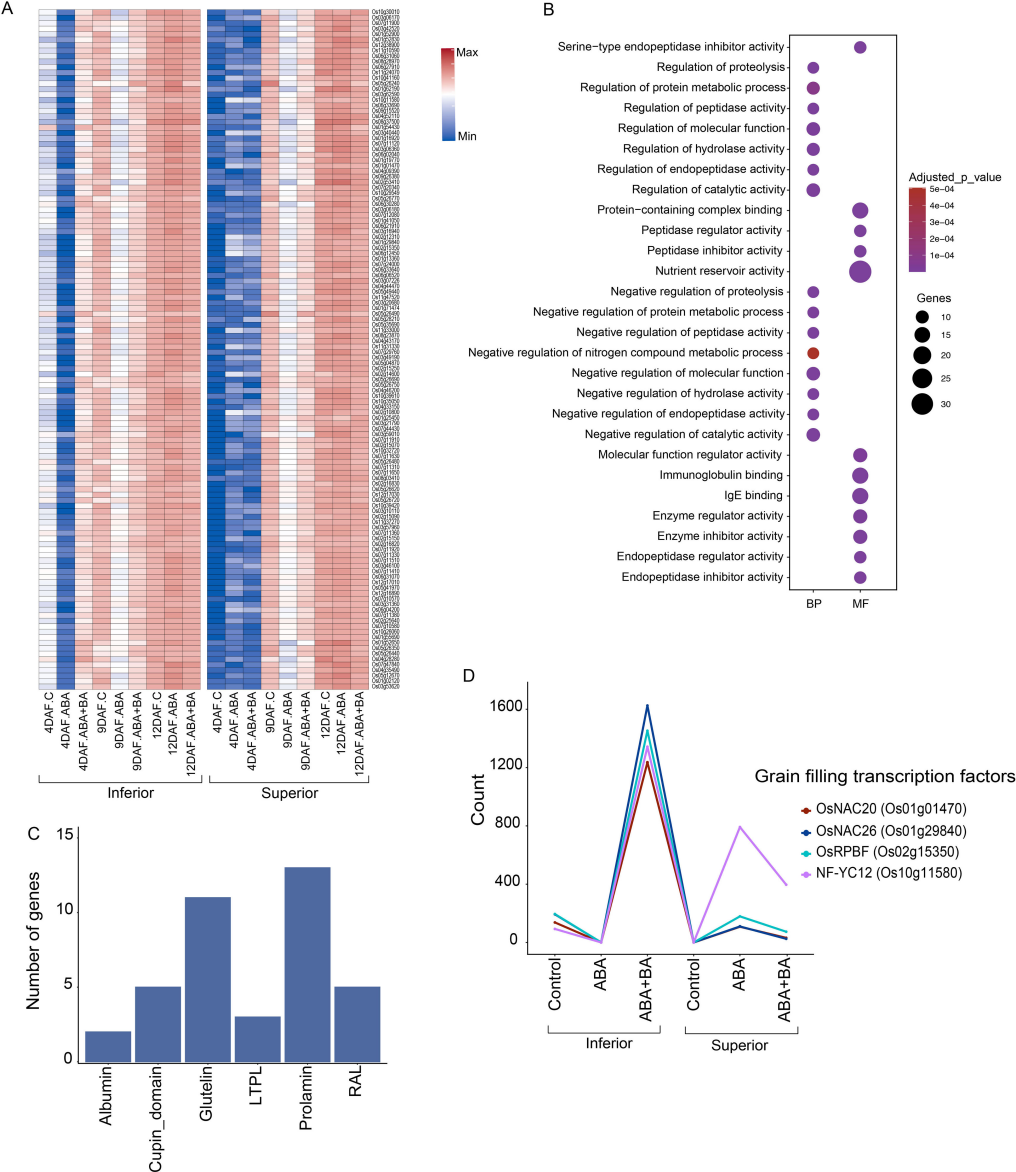
ABA and ABA+BA applications alter the regulation of starch and storage protein genes expression **(A)** Expression of 124 genes enriched in storage proteins **(B)** Gene ontology of cluster shows the enrichment of nutrient storage related genes **(C)** Distribution of protein storage coding genes family in the cluster **(D)** Induction pattern of key grain filling transcription factors in inferior and superior grains. BP, Biological process; MF, Molecular function; CC, Cellular component.

A difference in the cell cycle progression between superior and inferior grains next led us to examine the developmental stage of these grain types at 4 DAF. Our analysis yielded a group of 124 genes that have a higher expression in inferior grains compared to superior grains (**Figure 5A**). These genes were repressed under ABA but are induced by BA treatment in inferior grains at 4 DAF. In contrast, expression of these genes remains unaltered and lower in all conditions in superior grains at 4 DAF. This gene set is overrepresented in GO terms such as ‘nutrient reservoir activity’, ‘protein−containing complex binding’ and ‘immunoglobulin binding’ (**Figure 5B**). Several GO terms related to protein catabolic or anabolic process such as endopeptidase activity and regulation of proteolysis were also identified. From this gene set, 13 genes encode prolamin, 11 glutelin, five cupin domain, five Ras-like, three lipid transfer protein-like, and two albumin grain protein storage superfamilies (**Figure 5C**). Together with these storage proteins, starch biosynthesis is also regulated by exogenous BA application in inferior grains at 4 DAF as several TFs involved in starch synthesis such as *OsNAC20*, *OsNAC26 OsRPBF* and *NF-YC12* were specifically induced by BA in inferior grain (Huang et al., 2021) (**Figure 5D**). A similar pattern was observed for starch synthase *SSIIa* (*LOC_Os06g12450*) and grain filling *OsNAC025* (*LOC_Os11g31330*). We speculate that BA application alters the endogenous sugar level in the inferior grains as the sugar sensor, *OsNAC23*, showed a marked induction upon BA treatment, whereas its target *TREHALOSE-6-PHOSPHATE PHOSPHATASE1* (*TPP1*) was repressed at 4 DAF (Li et al., 2022). *OsNAC23*, being a positive sensor for sugar status, bridges sugar and trehalose-6-phosphate levels through the transcriptional repressor *TPP1*.

**Figure 6:**
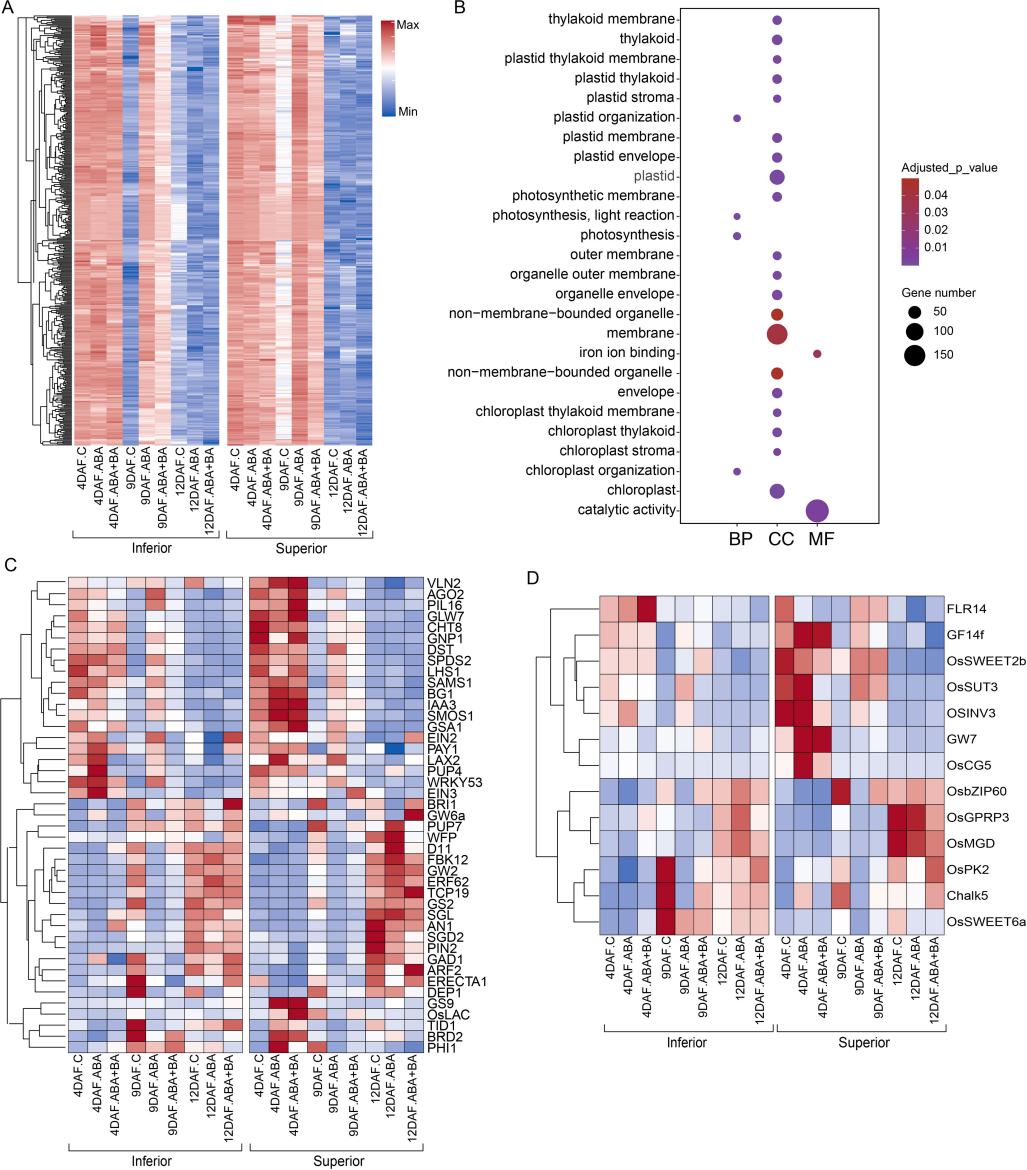
The expression of 417 genes is differentially regulated between superior and inferior grains at 9 DAF. **(A)** Temporal expression of superior and inferior grains under control conditions and in response to ABA and ABA+BA **(B)** Gene ontology enrichment of genes in **(A)**. **(C)** Expression pattern of previously characterized genes regulating grain size **(D)** Expression pattern of sucrose transporters grain quality related genes showing differential expression between superior and inferior grains.

Although variation in superior and inferior grains was suggested to be driven mainly by differential regulation of genes at 4 DAF (**Figure 2A**), our analysis revealed that genetic regulation at 9 DAF also contributes to grain-level phenotypic differences. We found that 417 genes were strongly suppressed in inferior grains at 9 DAF in control conditions, however, they had relatively higher expression in superior grains at the same developmental stage (**Figure 6A**). Notably, ABA and ABA+BA applications induced the expression of these genes in both grain types. GO terms indicate that several of these genes are plastid-localized and predominantly involved in various catalytic activities including roles in photosynthesis (**Figure 6B**). Of these genes, genetic studies showed the essential role of serine/threonine kinase *OsSTN8* in protein phosphorylation associated with PSII repair (Nath et al., 2013). PROTON GRADIENT REGULATION 5 (*LOC_Os08g45190*) is required for PSI cyclic electron transport (Nishikawa et al., 2012). Given the induction of these genes in inferior grains under phytohormone applications, which also improved the grain size, it is possible that the expression of these genes also influences mature grain size. Consistent with our hypothesis, we found genes that positively regulate grain size (**Figure 6C**). For instance, increasing the expression of a gibberellin biosynthesis pathway gene *GNP1* increases the grain number and yield (Wu et al., 2016). A cytokinin-O-glucosyltransferase gene *LOC_Os04g25440* is linked to the regulation of cytokinin production and in turn controlling grain production (Li et al., 2013).

Next, we analyzed sugar transport-related genes to identify whether there was any differential regulation between the superior and inferior grains. Among the SUT and SWEET family genes, we found significant differences in *OsSUT3*, *OsSWEET2b*, and *OsSWEET6a* expression between superior and inferior grains (**Figure 6D**). *OsSUT3* and *OsSWEET2b* had relatively higher expression in 4 DAF superior grains under control and ABA treatment than in inferior grains from the same treatment. In contrast, *OsSWEET6a* expression was highest in 9 DAF inferior samples under control conditions, wherein ABA and ABA+BA treatments suppressed its expression. Besides the sugar transporters, we also found differential expression of several genes between superior and inferior grains, such as *FERONIA-LIKE14* (He et al., 2023); *GF14f* (Lin et al., 2023); GW7 (Wang et al., 2015b); and *Chalky Grain 5* (Chandran et al., 2022), previously associated with grain quality (**Figure 6D**). These analyses indicate that besides improving the grain weight and fertility rate, ABA and ABA+BA regulate grain quality.

## Discussion

Efficient transportation and filling of photoassimilates to the cellularized endosperm are critical events during seed development, as the endosperm constitutes the major portion of the mature seed. While cytokinin levels regulate endosperm cell number and cell division activity, higher concentrations of ABA at the initiation of endosperm filling have been shown to be positively correlated with a more efficient grain filling process (Yang et al., 2002, 2022). Basic and applied research in the last four decades clearly demonstrates the role of ABA in initiating and playing a pivotal role during grain filling of key field crops such as wheat, barley, rice, corn, and soybean (King, 1976; Dewdney and Mcwha, 1978; Tietz et al., 1981; Schussler et al., 1984; Jones and Brenner, 1987; Kato et al., 1993). Besides the role in carbohydrate allocation, field plot ABA application also improved grain quality with enriched oil concentration in soybean (Travaglia et al., 2009). Therefore, exogenous applications of ABA and BA could be used to improve yield by improving the grain filling in inferior grains, which have poor filling quality due to the low concentrations and dynamic regulation of these phytohormones.

To explore strategies to maximize the grain filling efficiency of inferior grains, we investigated the effects of exogenous ABA and ABA+BA applications on rice SS and IS development, including the underlying molecular mechanisms. Inherently lower ABA combined with higher ethylene levels have been shown to cause insufficient grain filling in IS as these phytohormones at given ratios play a critical role in grain filling by enhancing the expression of starch metabolic genes (Wang et al., 2019). Our field study showed that along with superior panicles, the grain weight from inferior panicles is also significantly increased in response to both ABA and ABA+BA applications (**Figure 1A**). Besides grain weight, ABA and ABA+BA application improved grain fertility (**Figure 1B**). As a compound effect, the total grain yield and 1000-grain weight were significantly increased with ABA+BA, which conferred maximum yield potential (**Figures 1C, D**). These results show that field-level application of ABA+BA is a viable strategy to maximize rice yields.

An increase in grain yield and an inherent difference in the grain developmental response of SS and IS resulting from ABA and ABA+BA applications led us to examine the transcriptional changes induced by the phytohormone applications in both grain types. PCA of time-series RNASeq analysis at 4 DAF, 9 DAF and 12 DAF showed that although superior and inferior grains have a distinct transcriptomic signature under natural conditions, phytohormone application leads to an extensive transcriptional reprogramming at 4 DAF and 9 DAF compared to 12 DAF (**Figure 2A**). This pattern is not unexpected, because genes governing endosperm cellularization initiate as early as 24 HAF and critical factors such as endosperm cell numbers are determined before 9 DAF. On the other hand, grains at 12 DAF have entered the storage phase, wherein the endosperm cells accumulate starch, proteins, and other grain constituents. Superior and inferior grains in the control, ABA, and ABA+BA treatments revealed a core set of 7,326 genes that are either differentially expressed under various developmental stages or under phytohormone applications (**Figure 2C**). Superior and inferior grains shared only 55% of common DEGs (**Figure 2B**) and differential regulation of 28.7% and 16.2% of genes were specific to inferior and superior grains, respectively.

Given the improved grain filling in IS and SS along with extensive transcriptional reprogramming observed between superior and inferior grains, we sought to address the underlying genetic changes induced by ABA and ABA+BA in each grain developmental stage. Clustering analysis of DEGs revealed striking differences in the expression of genes in clusters 5, 8 and 9 between SS and IS at 4 DAF (**Figure 2C**). These clusters were populated with cell cycle regulatory genes, indicating that the primary target of ABA and ABA+BA on SS and IS is cell cycle regulation (Sun et al., 2015; Panda et al., 2018). We queried genes governing different stages of the mitotic cell cycle to determine how the cell cycle genes are regulated by ABA and ABA+BA. At the onset of mitosis, the CDK complex comprising CKs and CDKs drives through different cell cycle events (Qi and Zhang, 2020). Genes involved in the CDK complex at the G1 stage had similar expression patterns between superior and inferior grains. However, we found a strong induction of CDKB genes in superior grains (**Figure 4A**). At the G1 to S phase transition, retinoblastoma-related (RBR) protein is phosphorylated by CDKA/CYCD complex and RTR activates S phase E2Fs TFs, which in turn activates the downstream S phase genes involved in DNA replication and chromatin dynamics (Qi and Zhang, 2020). In contrast to G1 stage genes, the upregulation of two E2F TFs (*OsDEL1*; *LOC_Os02g50630*, *OsDEL2*; *LOC_Os06g13670*) in superior grains at 4DAF indicates that ABA and ABA+BA primarily act on G1 to S phase. Moreover, activation of DNA replication genes downstream of these TFs, involved in Pre-RC, replication fork assembly, fork progression, DNA polymerase, proof-reading activity and double strand break repairs further supports that ABA and ABA+BA application induce change in cell cycle progression at S phase uniquely in developing superior grains. While DNA replication genes are triggered, ABA and ABA+BA activated CDK inhibitor *OsSMR1* (*LOC_Os01g62584*) and its upstream regulator TF *SMOS1* (*LOC_Os05g32270*), which possibly causes a lower CDK activity in superior grains. Nevertheless, oscillation and lower a threshold of CDK activity have direct implications on endoreduplication (Larkins et al., 2001; Porter, 2008). It is noteworthy that overexpression of *SIM* can induce endoreduplication resulting in higher DNA content and has been proposed to inhibit only M-phase, unlike KRPs that block entry into both M and S phase (Kumar and Larkin, 2017). An unaltered expression of KRPs and activation of S phase genes therefore indicates that *OsSMR1* plays a critical role in switching cell cycle to endoreduplication upon phytohormone application. This speculation is consistent with the induction of other central players of cell cycle switches to endoreduplication under ABA+BA application in superior grains. For instance, WEE1 acting at the G2 to M phase induces endoreduplication and its overexpression has been shown to increase fruit size in tomatoes (Gonzalez et al., 2007). Besides WEE1, two activators of APC/C, *CDC20* and *CCS52*, are induced under ABA and ABA+BA. APC/C plays a major role in endoreduplication through irreversible degradation of cell cycle proteins.

Several lines of evidence indicating a cell cycle switch prompted us to examine the superior grains specific endoreduplication induced by phytohormones. Our analysis suggests that superior and inferior grains have a differential level of cytokinin metabolism and signaling based on the expression of the *OsCKX2* and multiple cytokinin response-regulator genes (*OsRR1*, *OsRR2*, *OsRR6*, *OsRR9*, *OsRR10* and *OsCKX2*) (**Figures 4C, D**). only the ABA+BA, and not the ABA application induces the expression of these genes specifically in inferior grains. In contrast, both ABA and ABA+BA suppress these genes in superior grains, suggesting a perturbation of cytokinin homeostasis in superior grains triggered by phytohormones. Besides ABA and ethylene, cytokinins also have been known to mediate grain development primarily through regulating endosperm cell division and proliferation activities (Yang et al., 2002, 2016). The contrasting expression patterns indicate that endogenous cytokinin is critical in phytohormone-mediated cell cycle regulation in superior grains.

Besides cell cycle genes, we found that 124 genes, primarily enriched with nutrient reservoir activity-related functions, are differentially expressed between SS and IS under control and phytohormone applications at 4 DAF (**Figure 5A**). These genes include storage protein-coding genes such as 13 prolamins, 11 glutelins, five cupin domains, five RAL, three LTPL, and two albumins (**Figure 5C**). Differential activity for these genes indicates that fine-tuning the storage protein accumulation in SS and IS contributes to improved grain filling. We also found a significant induction of starch synthesis TFs *OsNAC20*, *OsNAC26 OsRPBF* and *NF-YC12* in response to BA application in IS at 4 DAF, highlighting the role of BA in grain weight accumulation (**Figure 5D**). Moreover, BA evidently leads to an increase in endogenous sugar levels as the sugar sensor *OsNAC23* is highly induced upon BA application, whereas its target *TPP1* was repressed at 4DAF in IS. *OsNAC23* overexpressed varieties have been demonstrated to have a 13%–17% higher yield in elite rice varieties (Li et al., 2022).

Given the higher expression of storage protein and starch regulatory genes, we suggest that under natural conditions, inferior grains advanced through the cellularization stage and initiated the grain filling process at 4 DAF. A relatively short cellularization window in the inferior grains may be linked to the lower grain weight as the endosperm cellularization is positively linked to the grain weight. Moreover, suppression of these genes by ABA application corroborates the improved grain weight of inferior grains in our field study. Although most of the differences between SS and IS under control conditions and phytohormone application were identified at 4 DAF, a subset of 417 genes showed differential regulation at 9 DAF. These genes were suppressed in inferior grains at 9 DAF in control conditions, in contrast to their relatively higher expression in superior grains at the same developmental stage (**Figure 6A**). Notably, ABA and ABA+BA applications induced the expression of these genes in both grain types. Similarly, we found differential regulation of *OsSUT3*, *OsSWEET2b* and *OsSWEET6a* between superior and inferior grains, indicating their potential role in the grain development between these two grain types. The role of SUT and SWEET family genes in grain filling has been previously identified (Yang et al., 2018). Our analysis shows that these three transporters may regulate the sink capacity between superior and inferior grains. In addition, several characterized genes previously known to play a role in grain quality are also differentially regulated between superior and inferior grains, which underlines the genetic basis of grain quality difference between superior and inferior grains (**Figure 6D**).

Our analysis of ABA versus ABA+BA transcriptomes identified several genes that are largely induced at higher levels in inferior spikelets only after ABA+BA treatment at 9 DAF. These genes are inherently higher in untreated superior spikelets (**Figure 4E**) at 9 DAF. A subset of these genes was induced earlier in superior spikelet for both ABA and ABA+BA treatments. This suggests that enhanced improvement of yield parameters for inferior panicles under ABA+BA treatment relative to ABA treatment alone could be due to one or more of these genes. Further, improvement observed for superior panicles beyond ABA treatment could be attributed to earlier induction for the top cluster (4 DAF instead of 9 DAF). Alternatively, fine tuning of highly expressed genes in 9 DAF control superior spikelets in the bottom cluster (**Figure 4E**) by ABA+BA could possibly lead to yield enhancement beyond ABA treatment. This is plausible as ABA is primarily a stress hormone and increased levels of ABA from exogenous application and progression towards grain maturation may suppress gene(s) contributing towards higher yield. Adding BA to ABA treatment therefore could help mitigate the detrimental ABA-responsive genes. Although it is challenging to pinpoint the exact gene responsible for these yield enhancements from the dual application of ABA+BA, the high specificity of the two clusters provides a useful framework to narrow down the hypotheses that will be tested in future studies.

In summary, field level applications of ABA and BA significantly improved the grain yield by enhancing the grain filling efficiency of both superior and inferior grains, albeit by different mechanisms. In superior grains, transcriptome analysis showed that the induction of DNA replication and cell cycle inhibitory genes leads to activation of an endoreduplication switch in superior grains upon ABA and ABA+BA application at 4DAF, potentially resulting in higher yields. On the other hand, the induction of the cytokinin signaling and metabolic genes as well as grain storage related genes indicates the grain filling in inferior grains, while positive role of ABA-responsive transcriptome changes highlights the differential sensitivity of superior and inferior grains to phytohormones. Additional research is needed to understand the agronomic, physiological, and molecular aspects of the growing use of phytohormones to increase the yield and quality of field crops.

## Data Availability

The data presented in the study are deposited in the NCBI repository, accession number PRJNA1261674.

## Supplementary material

Supplementary Figure 1 Schematic diagram depicting the experimental design and hormone treatment.

Supplementary Table 1 Details of differentially expressed genes identified from samples pairwise comparisons at 4 DAF, 7 DAF and 10 DAF.
