# Supplementary figures and images for: Differential ABA sensitivity of superior and inferior rice grains is linked to cell cycle entry into endoreduplication
Source: Front Plant Sci. 2025 May 20;16:1585022. doi: 10.3389/fpls.2025.1585022 (PMC12129772; doi:10.3389/fpls.2025.1585022)

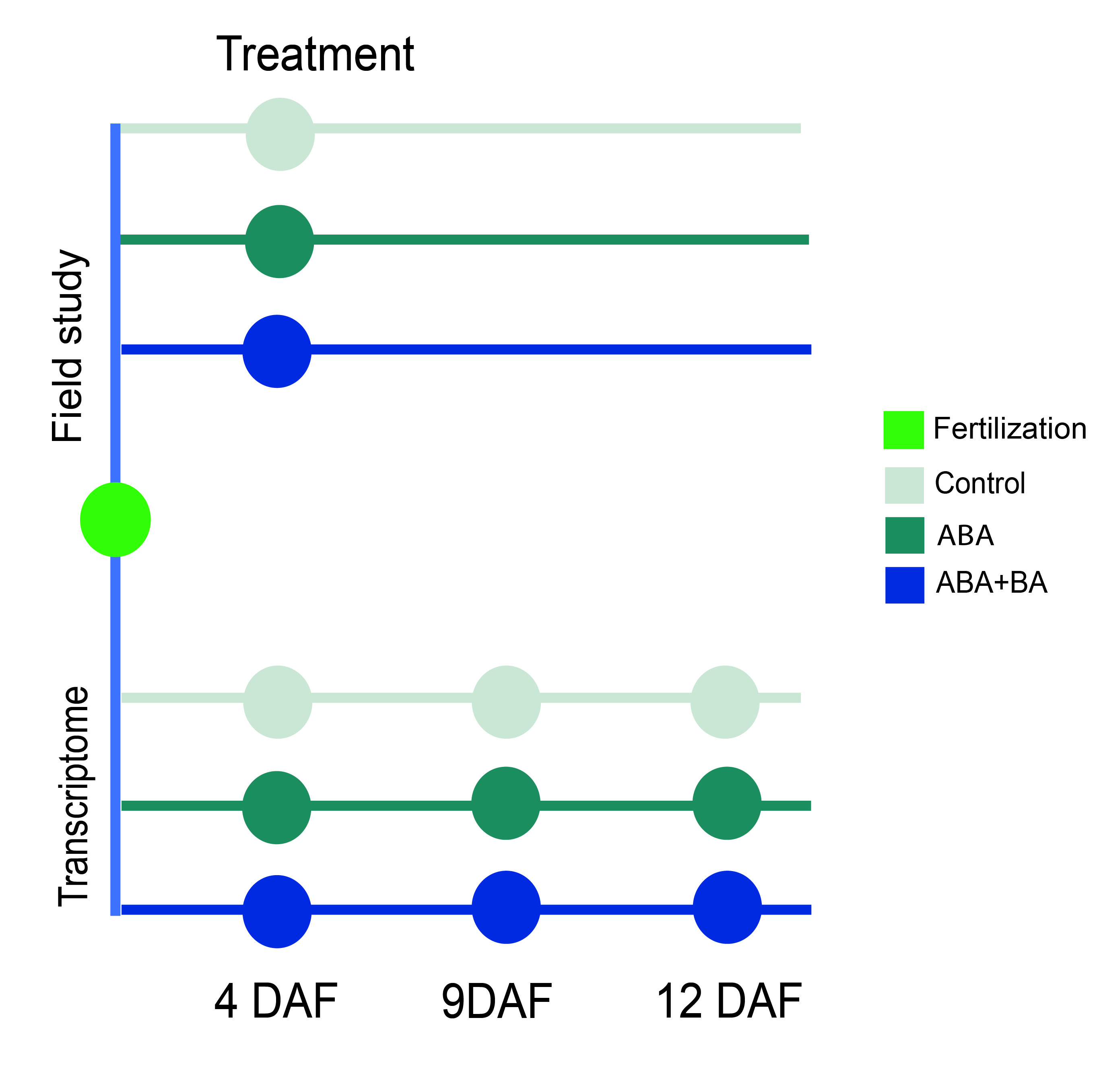

Supplement: Supplementary Figure 1 — Schematic diagram depicting the experimental design and hormone treatment. [file Image1.jpeg]
